# Breast cancer histopathological image classification using convolutional neural networks with small SE-ResNet module

**DOI:** 10.1371/journal.pone.0214587

**Published:** 2019-03-29

**Authors:** Yun Jiang, Li Chen, Hai Zhang, Xiao Xiao

**Affiliations:** College of Computer Science and Engineering, Northwest Normal University, 730070, Lanzhou Gansu, P.R.China; University of South Carolina, UNITED STATES

## Abstract

Although successful detection of malignant tumors from histopathological images largely depends on the long-term experience of radiologists, experts sometimes disagree with their decisions. Computer-aided diagnosis provides a second option for image diagnosis, which can improve the reliability of experts’ decision-making. Automatic and precision classification for breast cancer histopathological image is of great importance in clinical application for identifying malignant tumors from histopathological images. Advanced convolution neural network technology has achieved great success in natural image classification, and it has been used widely in biomedical image processing. In this paper, we design a novel convolutional neural network, which includes a convolutional layer, small SE-ResNet module, and fully connected layer. We propose a small SE-ResNet module which is an improvement on the combination of residual module and Squeeze-and-Excitation block, and achieves the similar performance with fewer parameters. In addition, we propose a new learning rate scheduler which can get excellent performance without complicatedly fine-tuning the learning rate. We use our model for the automatic classification of breast cancer histology images (BreakHis dataset) into benign and malignant and eight subtypes. The results show that our model achieves the accuracy between 98.87% and 99.34% for the binary classification and achieve the accuracy between 90.66% and 93.81% for the multi-class classification.

## 1 Introduction

Cancer is one of the leading cause of human death worldwide currently. For women, breast cancer-related deaths are higher compared to the other types of cancer-related deaths [1], and this type of cancer causes thousands of deaths each year worldwide [2]. It has been reported that the incidence rate of breast cancer ranges from 19.3 per 100,000 women in East Africa, to 89.7 per 100,000 women in Western Europe [3]. The number of new cases has continued to grow in recent years, and this number is expected to increase to 27 million in 2030 [4].

Breast cancer develops from breast tissue identified by lump in the breast and there are some changes in normal conditions [5]. Clinical screening includes mammography [6], breast ultrasound [7], biopsy [8] and other method. A biopsy [8] is the only diagnostic procedure that can definitely determine if the suspicious area is cancerous. The pathologists diagnose by visual inspection of histological slides under the microscope, which is considered as confirmatory gold standard for diagnosis [9]. However, the traditional manual diagnosis needs intense workload by experts with expertise. Diagnostic errors are prone to happen with the pathologists that have not enough diagnostic experience. It is shown that the use of Computer-aided diagnosis (CAD) [10] to automatically classify histopathological images can not only improve the diagnostic efficiency, but also provide doctors with more objective and accurate diagnosis results.

Deep Learning is a growing technology in the field of machine learning and it has got the attention of many researchers [11]. The Convolutional Neural Network (CNN) has achieved great success in a large-scale image and video recognition. Spanhol et al. [12] used AlexNet [13] to classify breast cancer pathology images for both benign and malignant categories. Their classification results are 6% higher than traditional machine learning classification algorithms. In [14], the author mentions that previously trained CNN reuse is used as a feature vector, and DeCAF features are extracted. Then, the DeCAF feature is used as an input to the classifier trained for the new classification task. It achieved an average of 84% accuracy on breast cancer case images. Kausik et al. [9] proposed a multiple instance learning (MIL) framework for CNN. They introduced a new pooling layer that helped to aggregate most informative features from patches constituting a whole slide, without necessitating inter-patch overlap or global slide coverage. An accuracy of about 88% was obtained on breast cancer case images. In [15], the author proposed a structured deep learning model for solving the subordinates of breast cancer, with the best classification result reaching 92.19%. In [16], the authors proposed that hybrid CNN unit could make full use of the local and global features of an image, so as to make a more accurate prediction. The author also introduces the bagging strategies and hierarchy voting tactic to help improve the performance of the classifier. Finally, 87.5% classification accuracy was obtained on the multiple classifications of breast cancer. Akba et al. [17] propose a novel regularisation technique for CNNs, and named it as the transitionmodule, which captures filters at multiple scales, and then collapses them via global average pooling to ease network size reduction from convolutional layers to FC layers. The transition module was able to adapt to a small data-set successfully by achieving accuracy rates of 91.9%. Wei et al. [18] proposed that the class and subclass labels of breast cancer should be used as a priori knowledge to suppress the feature distance of different breast cancer pathological images. At the same time, a data augmentation method was proposed, and the accuracy of the binary classifications was reached 97%. In [4], the author introduces two methods. The first method is based on the extraction of a set of handcrafted features encoded by two coding models (bag of words and locality constrained linear coding), and then support vector machines were trained for classificaiton. The second method is based on the design of convolutional neural networks. The experiment result shows that the convolutional neural network is superior to the classifier based on manual features. The accuracy of the two classifications is 96.15% and 98.33% respectively, and the accuracy of multi-classification is 83.31% and 88.23% respectively.

At present, automatic classification of pathological breast cancer images based on convolutional neural networks is still a very challenging problem. The specific reasons are as follows: (1) Due to the continuous deepening of the model, the number of parameters of CNN also increases rapidly, which easily leads to over-fitting of the model. To reduce the risk of over-fitting, a large number of breast cancer histopathological images are usually required as training data for training CNN. However, the cost of obtaining a large number of labeled breast cancer images is expensive. Therefore, in case of limited breast cancer image data, we need to reduce the model over-fitting risk from the perspective of reducing CNN parameters and using data augmentation methods [19]. (2) It is well known that various hyperparameters have a great influence on the performance of the CNN model, especially the learning rate. In the process of model training, it is often necessary to adjust the learning rate parameters to obtain better performance manually, which makes it difficult to apply the algorithm in real life applications by non-expert users [20]. In order to reduce the training parameters of CNN, we designed a lightweight convolutional neural network module based on the characteristics of breast cancer histopathological images, and designed a network for breast cancer histopathological image classification. Furthermore, in order to avoid complicated adjustment of learning rate, we designed a Gaussian error scheduler (ERF) to adjust the learning rate during training.

More specifically, the contributions of this paper are as followings: (1) To reduce the training parameters of the model and reduce the risk of model over-fitting, we designed a small SE-ResNet model based on the combination of residual module and Squeeze-and-Excitation block. Compared to the bottleneck SE-ResNet module and basic SE-ResNet module, the parameters of the small SE-ResNet module is reduced to 29.4% and 33.3%, respectively. (2) We propose a new learning rate scheduler named Gaussian error scheduler which can get excellent performance without complicatedly fine-tuning the learning rate. (3) We design a novel CNN network based on small SE-ResNet module, pooling layer, and fully connected layer. This model has been tested on the BreakHis dataset for binary classification and multi-class classification with competitive experimental results.

The remaining of this paper is organized as follows: in Section 2, we introduce the theory and structure of the small SE-ResNet network. Section 3 analyses the performance of the step schedule and proposes the ERF learning rate scheduler. Section 4 gives our experiment result, including the introduction to the BreakHis dataset, and the experiment settings. Finally, we make our conclusion in Section 5.

## 2 The small SE-ResNet

### 2.1 SE-ResNet

SE-ResNet [21] is built upon the convolution operation, which extracts informative features by fusing spatial and channel-wise information within local receptive fields. The core module of SE-ResNet is a combination of Squeeze-and-Excitation block (SE block) [21] and the residual block of the ResNet [19, 22], in the notation hereafter we call it SE-ResNet module.

According to the CNN theory, the convolutional operator can fit any transformation: **T**: **X** → **O**, $X\in R^{C^{\prime }\times H^{\prime }\times W^{\prime }}$, $O\in R^{C\times H\times W}$. For simplicity, in the notation hereafter we take **L** to be the last convolutional layer in the SE-ResNet module. Let **X**_0_ be the input of SE-ResNet module and $X=[x^{1},x^{2},...,x^{C^{\prime }}]$ be the input of **L**. Let **K** = [**k**_1_, **k**_2_, …, **k**_*C*_] be the filter kernels of **L**, where **k**_*c*_ refers to the parameters of the *c*-th filter. Then the output of **L** can be defined as **O** = [**o**_1_, **o**_2_, …, **o**_*C*_], where

$$\begin{array}{c} o_{c}=k_{c}*X=\sum_{i}^{C^{\prime }}k_{c}^{i}*x^{i}. \end{array}$$ (1)

Here * denotes convolution, and $k_{c}=\left[k_{c}^{1},k_{c}^{2},...,k_{c}^{C^{\prime }}\right]$ (bias terms are omitted), while $k_{c}^{i}$ is a 2D spatial kernel, and therefore represents a single channel of **k**_*c*_ which acts on the corresponding channel of **X**. Since the output is generated by the weighted summation of all channels of the input, channel dependencies are implicitly embedded in **k**_*c*_, but these dependencies are entangled with the spatial correlation captured by the filters [21]. SE block adaptively recalibrates channel-wise feature responses by explicitly modelling interdependencies between channels. Recalibrating the filter response involves two steps, squeeze and excitation [21]. The first step uses the global average pooling to squeeze the global spatial information into the channel descriptor [21]. Formally, a statistic $S=\left[s_{1},s_{2},...,s_{C}\right]\in R^{C}$ is generated by shrinking **O** through spatial dimensions *H* × *W*, where

$$\begin{array}{c} s_{c}=\frac{1}{H\times W}\sum_{i=1}^{H}\sum_{j=1}^{W}o_{c}\left(i,j\right). \end{array}$$ (2)

To make use of the information aggregated in the squeeze operation, we follow it with a second step which aims to capture channel-wise dependencies fully. We use a fully connected neural network with two hidden layers to automatically learn the nonlinear interaction and non-mutually-exclusive relationship between channels. The output of this fully connected neural network can be defined as

$$\begin{array}{c} \tilde{S}=\sigma \left(W_{2}\delta \left(W_{1}S\right)\right), \end{array}$$ (3)

where *δ* refers to the ReLU [23] function, *σ* refers to the Sigmoid function, $W_{1}\in R^{\bar{C}\times C}$, $W_{2}\in R^{C\times \bar{C}}$, $\bar{C}=C/r$, and *r* is the reduction ratio (default set 16). We can rewrite the **L** as $\tilde{O}=\left[{\tilde{o}}_{1},{\tilde{o}}_{2},...,{\tilde{o}}_{C}\right]$, where

$$\begin{array}{c} {\tilde{o}}_{c}=\tilde{s_{c}}\cdot o_{c}. \end{array}$$ (4)

Here $\tilde{s_{c}}\in \tilde{S}$ and ${\tilde{o}}_{c}$ refers to channel-wise multiplication between the feature map **o**_*c*_ and the scalar $\tilde{s_{c}}$. Following He et al. [22], shortcut connection (SR) is the connection which skip one or more layers and for gradients to propagate further and allow for efficient training of very deep nets. Assuming the input and output dimensions are the same, we can write the final output of SE-ResNet module as

$$\begin{array}{c} \tilde{X}=X_{0}+\tilde{O}. \end{array}$$ (5)

After training a batch of images per epoch, the cost function calculates the distance between the prediction and target results and obtains a loss value for updating the CNN weight by back-propagation. The gradient calculation formula for the SE-ResNet module is defined as

$$\begin{array}{c} \frac{\partial \tilde{X}}{\partial X_{0}}=\frac{\partial (X_{0}+\tilde{O})}{\partial X_{0}}=1+\frac{\partial \tilde{O}}{\partial X_{0}}. \end{array}$$ (6)

The shortcut connection ensures that the gradient is always greater than or equal to 1 in the back-propagation, which avoids the gradient disappearance problem of CNN. The most significant difference with the residual block is that the SE-ResNet module makes use of a global average pooling operation in the squeeze phase and two small fully connected layers in the excitation phase, followed by a channel-wise scaling operation.

### 2.2 Type of convolutions in SE-ResNet module

**Fig 1:**
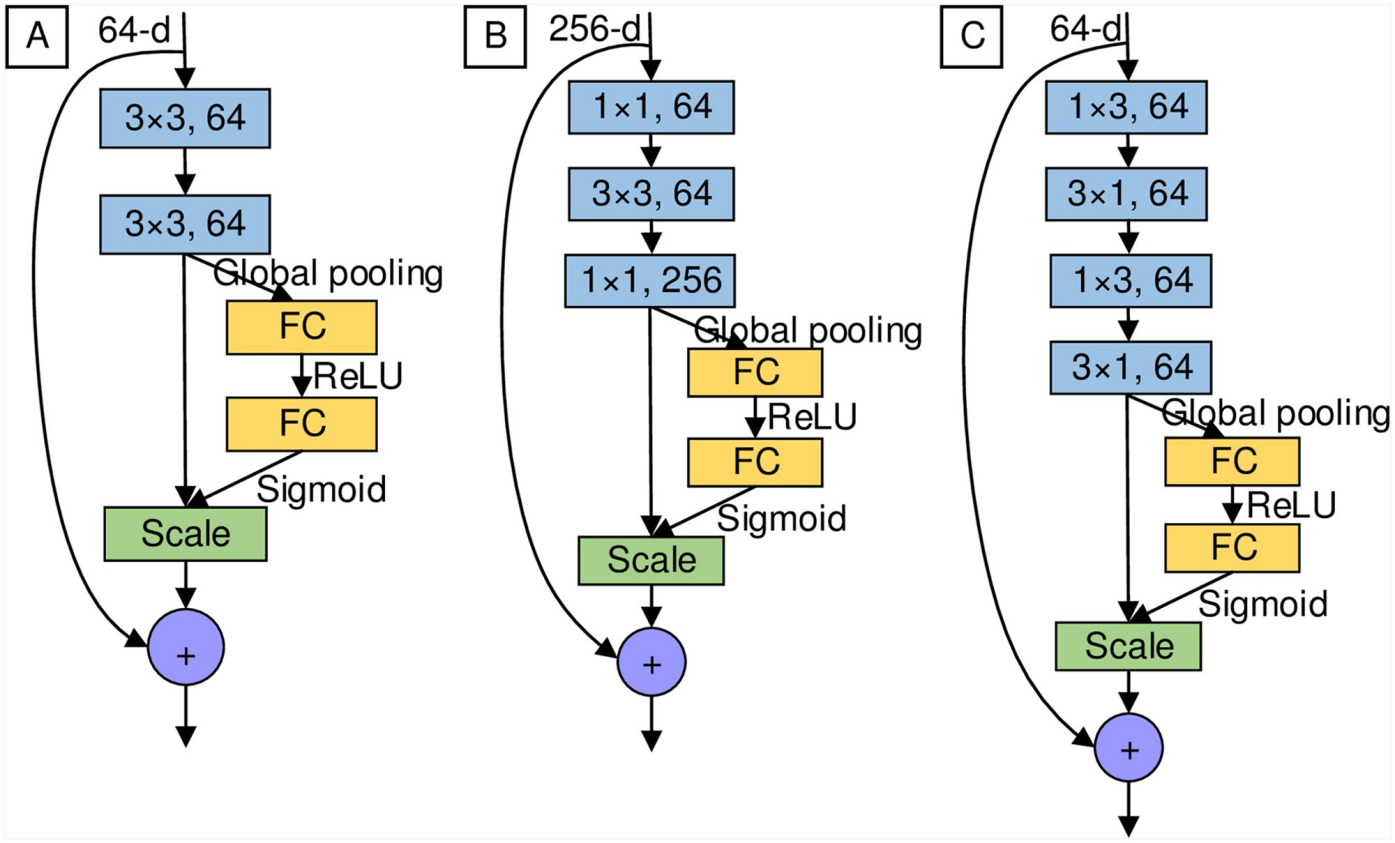
The SE-ResNet module architecture. (A) Basic SE-ResNet module. (B) Bottleneck SE-ResNet module. (C) Small SE-ResNet module.

Following He et al. [19, 22] and Hu et al. [21], the SE-ResNet module has two different structures:

A) basic SE-ResNet module—with two consecutive 3 × 3 convolutions with batch normalization and ReLU preceding convolution, and then it is combined with SE block: *conv*3 × 3—*conv*3 × 3—SE block (Fig 1A).
B) bottleneck SE-ResNet module—with one 3 × 3 convolution surrounded by dimensionality reducing and expanding 1 × 1 convolution layers, and then it is combined with SE block: *conv*1 × 1 − *conv*3 × 3—*conv*1 × 1—SE block (Fig 1B).

In this paper, we designed the small SE-ResNet module, a new SE-ResNet module to reduce the parameters of the network. In SE-ResNet module, there are two consecutive 1 × 3 and 3 × 1 convolutions with batch normalization, and ReLU preceding convolution, then combined with SE block: *conv*1 × 3—*conv*3 × 1—*conv*1 × 3—*conv*3 × 1—SE block (Fig 1C). We only consider the total number of parameters in the convolutional layers. The total number of parameters for one convolutional layer is:

$$\begin{array}{c} T(conv)=C\times H\times W\times K, \end{array}$$ (7)

where *C* × *H* × *W* is the size of the kernel and *K* is the number of kernels. Then the number of parameters for the three modules in Fig 1 could be got by the following formulas:

$$\begin{array}{c} T\left(basic\right)=\left(64\times 3\times 3\times 64\right)\times 2=18\times 64^{2}, \end{array}$$ (8)

$$\begin{array}{c} T\left(bottleneck\right)=256\times 1\times 1\times 64+64\times 3\times 3\times 64+64\times 1\times 1\times 256=17\times 64^{2}, \end{array}$$ (9)

$$\begin{array}{c} T\left(small\right)=\left(64\times 1\times 3\times 64\right)\times 2+\left(64\times 3\times 1\times 64\right)\times 2=12\times 64^{2}. \end{array}$$ (10)

Compared with the bottleneck SE-ResNet module and basic SE-ResNet module, the parameters of small SE-ResNet module are reduced by about 29.4% and 33.3%, respectively. To further evaluate the classification performance of different types of SE-ResNet modules, we consider the performance of five SE-ResNet architectures on Cifar image dataset [24]. Since the image size of Cifar is only 32 × 32, we make some change to the original SE-ResNet architecture as: in conv1, the filter kernels size is changed from 7 × 7 to 3 × 3 and stride is changed from 2 to 1, and removed the first max-pooling layer in conv2. We describe the architectures of SE-ResNet in Table 1.

**Table 1:** SE-ResNet architectures for Cifar. Building modules are shown in brackets, with the numbers of modules stacked. Downsampling is performed by conv3_1, conv4_1, and conv5_1 with a stride of 2.

Name	Output size	SE-ResNet-18	SE-ResNet-26	SE-ResNet-34	SE-ResNet-50	SE-ResNet-66
conv1	32 × 32	7 × 7, 64, stride 2, 3 × 3, 64, stride 1				
conv2_x	32 × 32	3 × 3 max pool, stride 2				
		$[\begin{array}{c} 3\times 3,64\\ 3\times 3,64\\ fc,[4,64] \end{array}]\times 2$	$[\begin{array}{c} 1\times 1,64\\ 3\times 3,64\\ 1\times 1,256\\ fc,[16,256] \end{array}]\times 2$	$[\begin{array}{c} 1\times 3,64\\ 3\times 1,64\\ 1\times 3,64\\ 3\times 1,64\\ fc,[4,64] \end{array}]\times 2$	$[\begin{array}{c} 1\times 1,64\\ 3\times 3,64\\ 1\times 1,256\\ fc,[16,256] \end{array}]\times 3$	$[\begin{array}{c} 1\times 3,64\\ 3\times 1,64\\ 1\times 3,64\\ 3\times 1,64\\ fc,[4,64] \end{array}]\times 3$
conv3_x	16 × 16	$[\begin{array}{c} 3\times 3,128\\ 3\times 3,128\\ fc,[8,128] \end{array}]\times 2$	$[\begin{array}{c} 1\times 1,128\\ 3\times 3,128\\ 1\times 1,512\\ fc,[32,512] \end{array}]\times 2$	$[\begin{array}{c} 1\times 3,128\\ 3\times 1,128\\ 1\times 3,128\\ 3\times 1,128\\ fc,[8,128] \end{array}]\times 2$	$[\begin{array}{c} 1\times 1,128\\ 3\times 3,128\\ 1\times 1,512\\ fc,[32,512] \end{array}]\times 4$	$[\begin{array}{c} 1\times 3,128\\ 3\times 1,128\\ 1\times 3,128\\ 3\times 1,128\\ fc,[8,128] \end{array}]\times 4$
conv4_x	8 × 8	$[\begin{array}{c} 3\times 3,256\\ 3\times 3,256\\ fc,[16,256] \end{array}]\times 2$	$[\begin{array}{c} 1\times 1,256\\ 3\times 3,256\\ 1\times 1,1024\\ fc,[64,1024] \end{array}]\times 2$	$[\begin{array}{c} 1\times 3,256\\ 3\times 1,256\\ 1\times 3,256\\ 3\times 1,256\\ fc,[16,256] \end{array}]\times 2$	$[\begin{array}{c} 1\times 1,256\\ 3\times 3,256\\ 1\times 1,1024\\ fc,[64,1024] \end{array}]\times 6$	$[\begin{array}{c} 1\times 3,256\\ 3\times 1,256\\ 1\times 3,256\\ 3\times 1,256\\ fc,[16,256] \end{array}]\times 6$
conv5_x	4 × 4	$[\begin{array}{c} 3\times 3,512\\ 3\times 3,512\\ fc,[32,512] \end{array}]\times 2$	$[\begin{array}{c} 1\times 1,512\\ 3\times 3,512\\ 1\times 1,2048\\ fc,[128,2048] \end{array}]\times 2$	$[\begin{array}{c} 1\times 3,512\\ 3\times 1,512\\ 1\times 3,512\\ 3\times 1,512\\ fc,[32,512] \end{array}]\times 2$	$[\begin{array}{c} 1\times 1,512\\ 3\times 3,512\\ 1\times 1,2048\\ fc,[128,2048] \end{array}]\times 3$	$[\begin{array}{c} 1\times 3,512\\ 3\times 1,512\\ 1\times 3,512\\ 3\times 1,512\\ fc,[32,512] \end{array}]\times 3$
fc	1 × 1	global average pool, 10d or 100d fc, softmax				

Each SE-ResNet is trained with the same optimization schemes. During training on Cifar, we follow standard practice and perform data augmentation. The optimization is performed using SGD with a momentum of 0.9 and a mini-batch size of 128. The initial learning rate is set to 0.1 and decreased by a factor of 5 after each of the 60, 120, and 160 epochs. We didn’t fine-tune the hyper-parameters of the network very carefully. Each experiment was repeated for 3 times, and the averaged results are reporeted here as the final result in Table 2.

**Table 2:** Experimental results of different SE-ResNet architectures on Cifar.

Method	Module	Cifar-10			Cifar-100		
		#params	#model size	Accuracy	#params	#model size	Accuracy
**SE-ResNet-18**	basic	11, 272*K*	90.4Mb	94.85 ± 0.14	11, 312*K*	90.8Mb	75.86 ± 0.22
**SE-ResNet-26**	bottleneck	15, 383*K*	123.4Mb	93.90 ± 0.18	15, 567*K*	124.8Mb	75.40 ± 0.27
**SE-ResNet-34**	small	8, 145*K*	65.6Mb	94.79 ± 0.17	8, 191*K*	65.9Mb	75.81 ± 0.13
**SE-ResNet-50**	bottleneck	26, 100*K*	209.4Mb	94.67 ± 0.09	26, 285*K*	210.8Mb	78.02 ± 0.25
**SE-ResNet-66**	small	14, 986*K*	120.7Mb	95.26 ± 0.18	15, 033*K*	121.1Mb	77.02 ± 0.20
**BHCNet-3**	small	198*K*	2.1Mb	92.30 ± 0.14	204*K*	2.2Mb	68.36 ± 0.23
**BHCNet-6**	small	401*K*	4.2Mb	93.18 ± 0.14	407*K*	4.2Mb	70.33 ± 0.25

In Table 2, firstly, it is shown that SE-ResNet-34 has 27.74% fewer parameters than SE-ResNet-18, but the accuracy is reduced only by less than 0.06% on Cifar. Secondly, SE-ResNet-66 not only has fewer parameters than SE-ResNet-26 but also performs with higher accuracy. Thirdly, SE-ResNet with the bottleneck SE-ResNet module may not be suitable for Cifar-10 classification tasks, probably due to the conv4_x feature map explosion, and there are too many parameters of the last layer of fully connected layers. Finally, the parameters of SE-ResNet-66 are 42.81% less than SE-ResNet-50, but the accuracy of Cifar-100 is reduced by 1%, which we think is acceptable.

### 2.3 Network for breast cancer histopathology image classification

**Fig 2:**
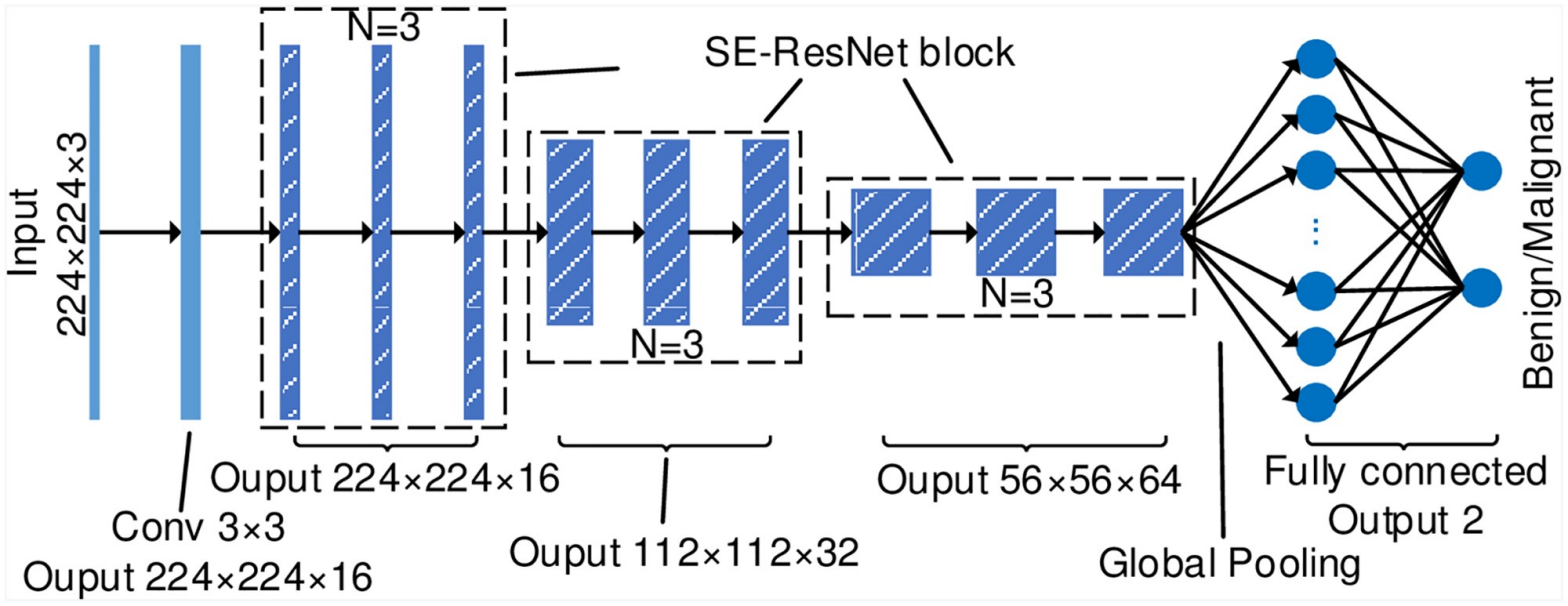
The BHCNet-3 architecture for the benign and malignant classification of breast cancer histopathological images.

As we know, the CNN model contains a high capacity that can represent various functions while not requiring extracting features manually. Therefore, we use CNN to automatically extract the characteristics of breast cancer histopathology images and take full advantage of them for classification. We design a novel CNN architecture for the classification of breast cancer histopathology images using the small SE-ResNet module, which is named as the breast cancer histopathology image classification network (BHCNet). BHCNet includes one plain convolutional layer, three SE-ResNet blocks, and one fully connected layer. Each SE-ResNet block is stacked by *N* small SE-ResNet modules, which is denoted as BHCNet-*N* in this paper. When *N* = 3, the BHCNet architecture is shown in Fig 2. The BHCNet-3 model has 198k parameters and the model size is just 2.1Mb, which is implemented by the Keras [25] framework. The experimental results of BHCNet-3 and BHCNet-6 on Cifar are shown in Table 2. The BHCNet has very few parameters and can achieve measurable competitive results.

## 3 Gauss error scheduler

### 3.1 The performance analysis of step scheduler

The core idea of the Stochastic Gradient Descent (SGD) algorithm [26] is to select a sample randomly to calculate the gradient, and to update the parameters during each training process. The gradient of the loss function determines the updating direction of SGD. The parameter *θ*_*t*_ of time *t* is updated by *θ*_*t*_ = *θ*_*t*−1_ − *lr*_*t*_∇_*θ*_ *L*, in which *L* is the loss function, ∇_*θ*_ *L* is the gradient of *L*, and *lr*_*t*_ is the learning rate at time *t*. While stochastic gradient is simple and effective, it requires adjusting the model hyper-parameters carefully, especially, the learning rate used in optimization. A larger learning rate will cause CNN training to diverge, while a smaller learning rate will make CNN training converge slowly. Usually, researchers need to do experiment with various sizes of the learning rate to make the network converge faster and get better performance.

The step scheduler is the most used for scheduling the SGD learning rates. On ImageNet, the heuristic which AlexNet [13] followed is learning rate initialized with 0.01, and then the learning rate is divided by 10 when the validation error rate stop improving with the current learning rate, and reduce three times prior to termination. For ResNet, it starts with a learning rate of 0.1 and divide it by 10 at 30 and 60 epochs. On Cifar, ResNet begins with a learning rate of 0.1, divide it by 10 at 32k and 48k iterations and terminate training at 64k iterations but Wide ResNet [27] using learning rate dropped by 0.2 at 60, 120 and 160 epochs. It can be seen that the step scheduler used by different CNN architecture of the same dataset is different and the step scheduler used by the same CNN architecture of different datasets is also different.

The step scheduler is a very flexible method, which can be summarized as four hyper-parameters that need to be fine-tuned carefully: initial learning rate, training epochs, decay stages, and decay rate. We follow the cutout experiment by DeVries et al. [28] to discuss the performance of different step scheduler on Cifar-10. Following DeVries et al. [28], we train ResNet with 18 layers (denote as ResNet-18) and train for 200 epochs with batches of 128 images using SGD, Nesterov momentum of 0.9, and weight decay of 5e-4. The baseline step scheduler start with a learning rate of 0.1 and divide it by 5 after each of the 60, 120, and 160 epochs (denote as step-baseline). For comparison, we have designed some new step schedulers, denote as step-*R*. The learning rate is set to 0.1 initially and is divided by 10 at *E*_1_ and *E*_2_ epochs. Let $R=\frac{E_{1}}{E_{2}}\in \left(0,1\right)$ be the ratio of *E*_1_ and *E*_2_. Let *E*_1_ = ⌊*R* × *E*_2_⌋ and $E_{2}=\lfloor \frac{200}{1+R}\rfloor$.

**Fig 3:**
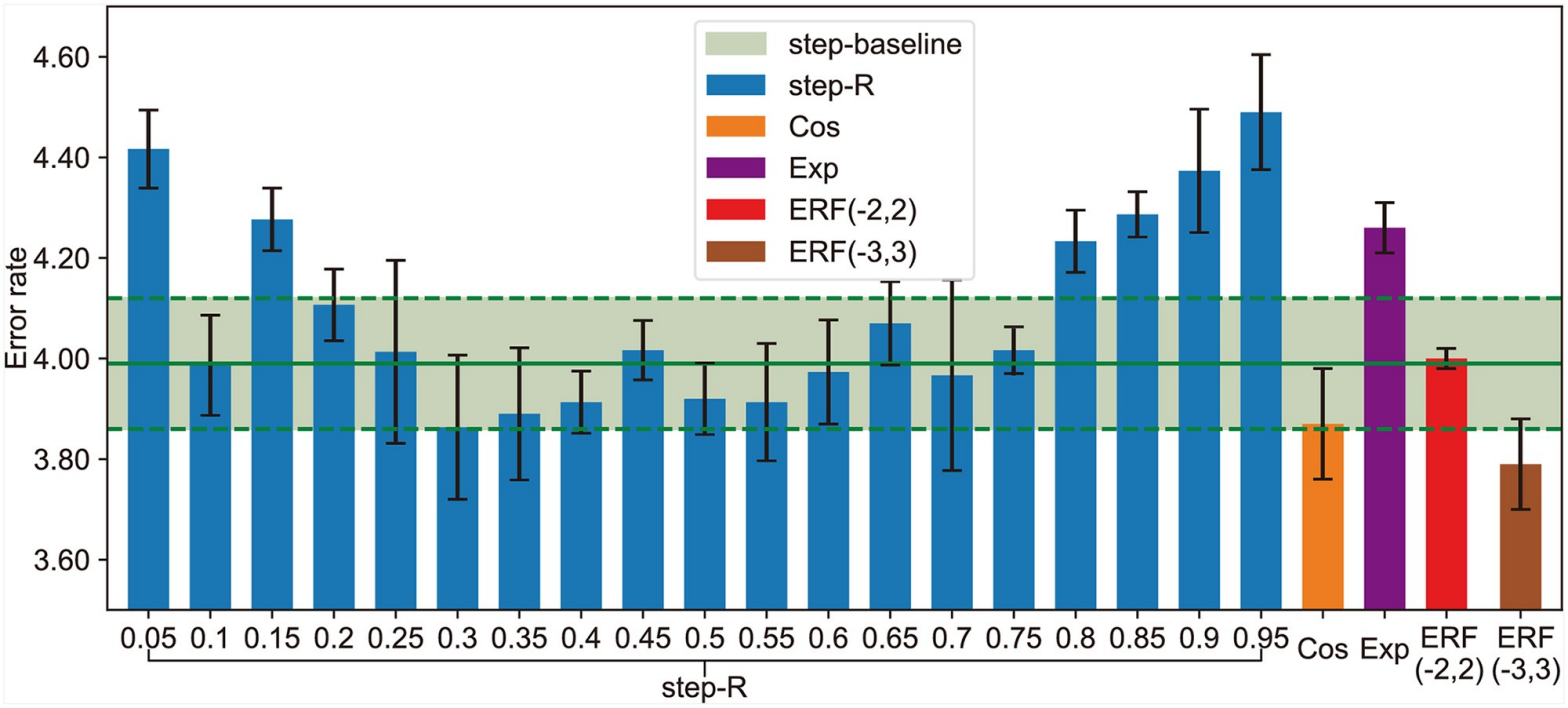
Comparison of experimental results of different learning rate schedulers on Cifar-10.

In the experiment by DeVries et al. [28], the cutout uses the baseline scheduler step to achieved an error rate of 3.99±0.13 on Cifar-10. The experimental results of different ratios *R* compared with the baseline are shown in Fig 3. We repeat each experiment for three times and report their average results. In this experiment, when *R* = 0.3 (denote as step-*R* = 0.3), the performance of step-*R* = 0.3 is better than step-baseline and achieves an error rate of 3.86±0.14 on Cifar-10 and have *E*_1_ = 45, *E*_2_ = 153, which is entirely different with step-baseline. Although we find a better result than the baseline step scheduler on Cifar-10, we are not sure that it is the best step scheduler for this experiment. This result implies that it is important for a step scheduler to choose the hyper-parameters for training.

### 3.2 Motivation

The primary motivation for Gauss error scheduler comes from the problem of fine-tuning the learning rate for BHCNet. In the training process of BHCNet, we increase the training epochs from 200 to 300, because in our pilot experiment, it is shown that training with 300 epochs can achieve better results than with 200 epochs. Most of the previous research experience used the step scheduler with 200 training epochs, however, to the best of our konwledge, there is no 300 epochs step scheduler for us to use. So we explore many different step schedulers to train BHCNet and the final classification accuracy increases from 97% to 98%. The progress of fine-tuning the step scheduler takes up a lot of time. Therefore, we further use the cosine scheduler [20] and exponential scheduler [29] to train BHCNet. According to experiments result, we find that the performance of the cosine scheduler and exponential scheduler is not as good as that of step scheduler for BHCNet, which may be due to the learning rate decayed too fast. The intrinsic random motion across gradient steps prevents the optimizer from reaching any of the sharp basins along its optimization path when the learning rate is large. The model tends to converge into the closest local minimum when the learning rate is small. Therefore, we want to propose a flexible learning rate scheduler, which consists of three stages. In the first stage, it provides a large learning rate for CNN and avoids CNN reaching the sharp basin. In the second stage, it attenuates the learning rate and does not require us to select the decay stages manually. In the third stage, it provides a small learning rate that the CNN converge to the closest local minimum. Our goal is that the new learning rate scheduler can compete with the carefully fine-tuned step scheduler.

### 3.3 Gauss error scheduler

The Gaussian error function [30] is a non-basic function, which is widely used in probability theory, statistics, and partial differential equations. It is defined as

$$\begin{array}{c} erf\left(x\right)=\frac{2}{\sqrt{\pi }}\int_{0}^{x}\exp\left(-t^{2}\right)\;dt. \end{array}$$ (11)

**Fig 4:**
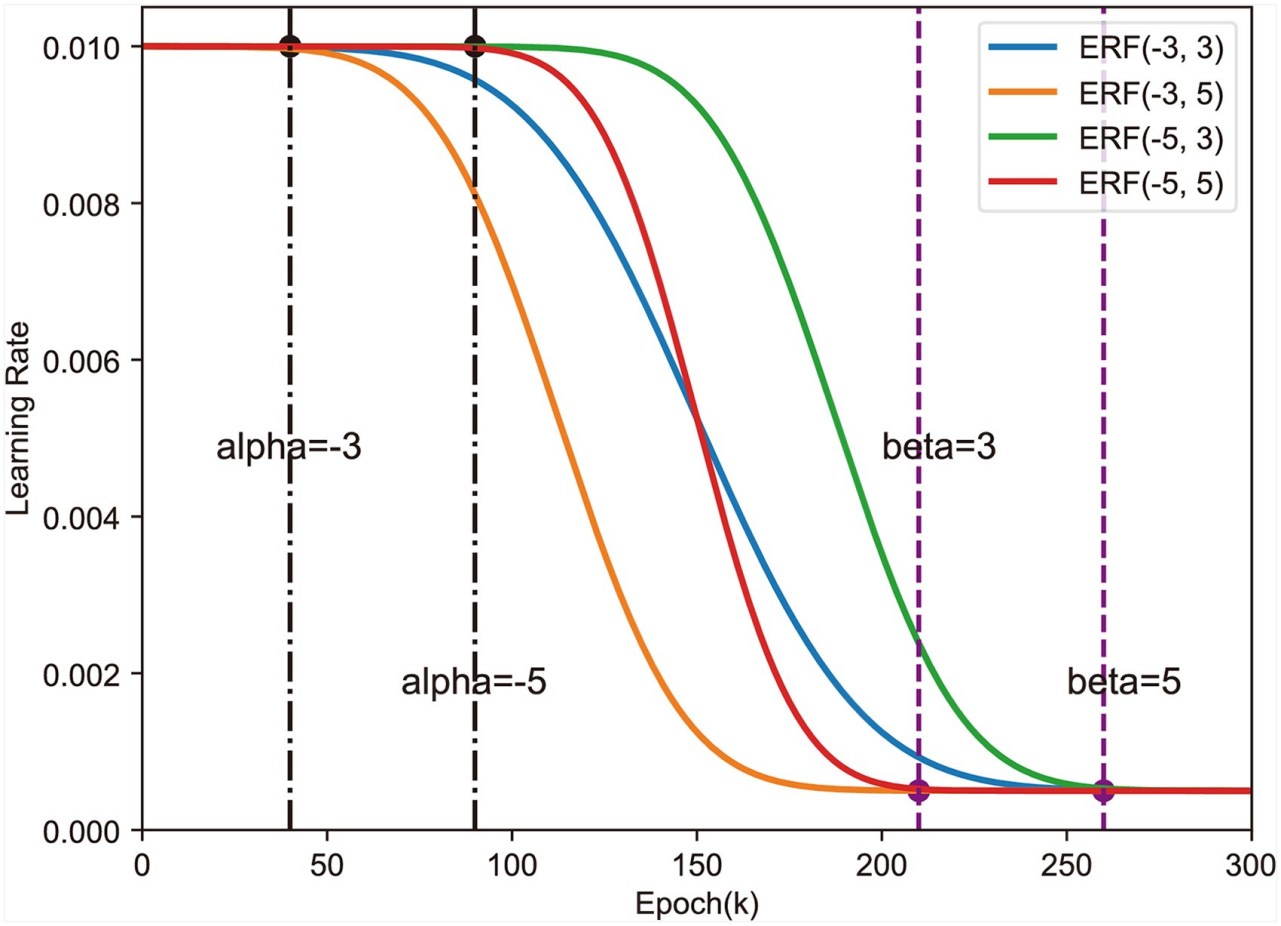
Gaussian error scheduler with different *α* and *β*.

According to the properties of the Gaussian error function, we design a Gauss error scheduler (denote as ERF), which controls the learning rate according to:

$$\begin{array}{c} ERF\left(\alpha ,\beta \right)=lr_{min}+\frac{lr_{max}-lr_{min}}{2}\left(1-erf\left(\frac{e(\beta -\alpha )}{E}+\alpha \right)\right), \end{array}$$ (12)

where *lr*_*max*_ denotes the maximum learning rate, *lr*_*min*_ denotes the minimum learning rate, *E* denotes the total number of epochs, *e* ∈ (0, *E*] denotes the current epoch, *α* denotes a negative integer, and *β* denotes a positive integer. The learning rate curves of Gaussian error scheduler with different *α* and *β* is shown in Fig 4. The running time that CNN requires by training at the initial learning rate is determined by *α*. The larger |*α*| is the longer running time CNN requires by training with using the *lr*_*max*_ learning rate. The time that CNN trains at the small learning rate is determined by *β*. The larger *β* is the longer time CNN needs to train using the *lr*_*min*_ learning rate. The ratio |*α*|/|*β*| determines the learning rate decay rate.

When the learning rate is close to zero, the noise will dominate the update of the CNN weights. It is inappropriate to set the learning rate approaching to zero in the later period, which can cause fluctuations and declines in the test accuracy during the final period. So we set the *lr*_*min*_ parameter of Gaussian error scheduler by ensuring that the learning rate does not close to zero, and we think the *lr*_*min*_ does not require fine-tuning very carefully. In this paper, *lr*_*min*_ is set to the minimum learning rate of step scheduler and *lr*_*max*_ is set to the initial learning rate of step scheduler.

Gauss error scheduler can be easily combined with SGD and optimization algorithms. The Nesterov Momentum SGD with Gaussian error scheduler algorithm is shown in Algorithm 1.

**Algorithm 1** Nesterov Momentum SGD with Gaussian error scheduler

**Require:** Maximum learning rate *l*_*max*_, minimum learning rate *l*_*min*_, Gaussian error scheduler parameter *α* and *β*, momentum parameter *m*.

**Require:** Initial parameter *θ*, initial velocity *v*, initial epochs *E*.

 **for** *e* = 1 to *E* **do**

  Sample a minibatch of m examples from the training set {*x*(1), …, *x*(*m*)} with corresponding labels *y*(*i*).

  Apply interim update: $\tilde{\theta }\leftarrow \theta +mv$

  Compute gradient (at interim point): $g\leftarrow \frac{1}{m}\nabla_{\tilde{\theta }}\sum_{i}L\left(f\left(x^{\left(i\right)};\tilde{\theta }\right),y^{\left(i\right)}\right)$

  Compute learning rate: $\varepsilon =l_{min}+\frac{l_{max}-l_{min}}{2}\left(1-erf\left(\frac{e(\beta -\alpha )}{E}+\alpha \right)\right)$

  Compute velocity update: *v* ← *αv* − *εg*

  Apply update: *θ* ← *θ* + *v*

 **end for**

### 3.4 Performance analysis

To further evaluate the performance of the Gaussian error scheduler, we use four different learning rate schedulers for experiment on the cutout. For the step scheduler, we use two different solutions: step-baseline and step-*R* = 0.3(Please refer to Section 3.1). For the Gauss error scheduler, we set *lr*_*max*_ = 0.1 and *lr*_*min*_ = 0.0001, and use two different solutions, ERF(-2, 2) and ERF(-3, 3). Following in [20], the cosine scheduler is defined as:

$$\begin{array}{c} \begin{array}{c} Cos=lr_{min}+\frac{lr_{max}+lr_{min}}{2}\left(1+\cos\left(\frac{\pi \times e}{E}\right)\right), \end{array} \end{array}$$ (13)

where *lr*_*max*_ is the maximum learning rate, *lr*_*min*_ is the minimum learning rate (defaultly *lr*_*min*_ = 0), *E* is the total number of training epochs, and *e* ∈ (0, *E*] is the current epoch. In this experiment, the parameters of the cosine scheduler are set to: *lr*_*max*_ = 0.1 and *lr*_*min*_ = 0. Following in [29], the exponential scheduler is defined as:

$$\begin{array}{c} Exp=lr_{0}\times \lambda^{e}, \end{array}$$ (14)

where *lr*_0_ is the initial learning rate, *e* ∈ (0, *E*] is the current epoch, and *λ* ∈ [0, 1] is a discount factor. In this experiment, the parameters of the exponential scheduler are set to: *lr*_0_ = 0.1 and *λ* = 0.98. The experiment results is shown in Table 3.

**Table 3:** The error rate of different schedulers on Cifar with ResNet-18 and cutout.

Scheduler	Cifar-10	Cifar-100
**step-baseline** [28]	3.99 ± 0.13	21.96 ± 0.24
**step -*R* = 0.3**	3.86 ± 0.14	22.56 ± 0.13
**Cos**	3.87 ± 0.11	21.42 ± 0.10
**Exp**	4.26 ± 0.05	22.10 ± 0.16
**ERF(-2,2)**	4.00 ± 0.02	**21.06** ± **0.10**
**ERF(-3,3)**	**3.79** ± **0.09**	21.53 ± 0.14

As shown in Table 3, the addition of Gauss error scheduler to the ResNet18 and cutout increased their accuracy on Cifar by between 0.2 to 0.9 percentage points. Compared with the step scheduler, the Gaussian error scheduler has fewer parameters that require to be fine-tuned, and it achieves better performance. The step-*R* = 0.3 achieved better results than the step-baseline on Cifar-10, but the results on Cifar-100 were 0.6% lower than that of step-baseline. ERF(-3,3) achieved better results than the step-baseline and step-*R* = 0.3 on Cifar. Compared with the cosine scheduler, the Gaussian error scheduler is more flexible and achieves better performance.

## 4 Experimental results

### 4.1 Materials

**Fig 5:**
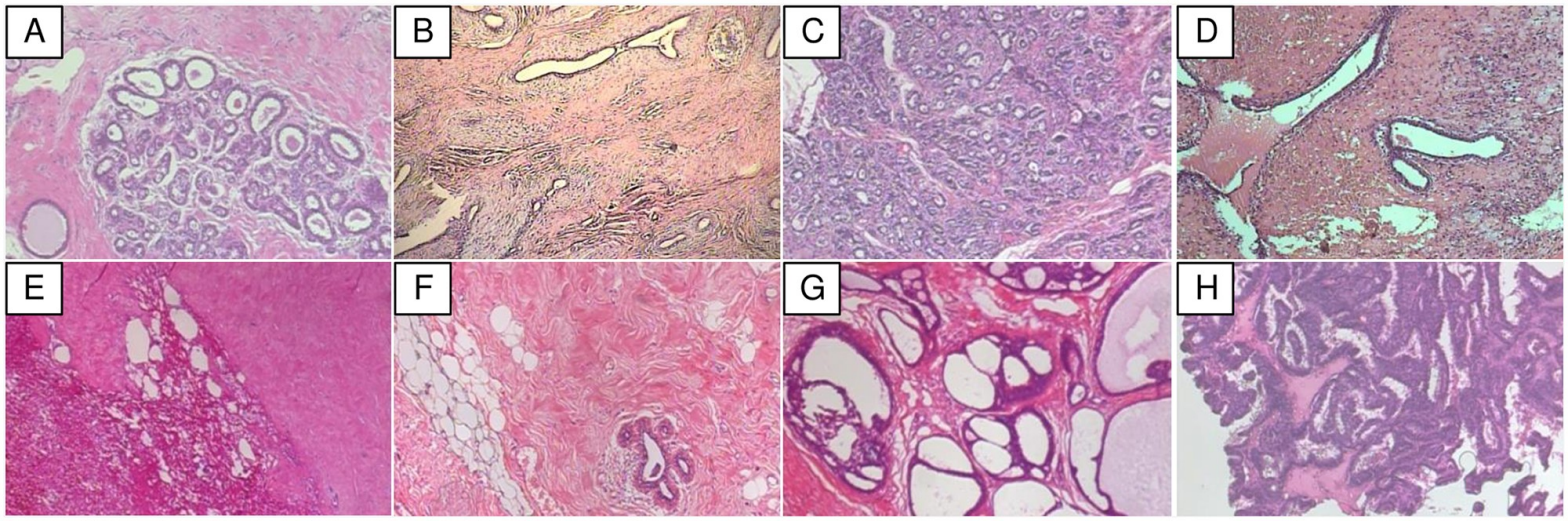
Image samples from the BreaKHis 40× dataset. (A) Adenosis, (B) Fibroadenoma, (C) Tubular Adenoma, Phyllodes Tumor, (D) Ductal Carcinoma, (E) Lobular Carcinoma, (F) Mucinous Carcinoma, (G) Papillary Carcinoma.

To analyze the performance of the BHCNet and Gauss error scheduler, we test them on Breast Cancer Histopathological Image (BreaKHis) [31]. The dataset contains 7,909 microscopic images (2,480 images for benign breast tumors and 5,429 images for malignant breast tumors with various magnification, including 40×, 100×, 200×, and 400×). Each image is encoded in 700 × 460 pixels by PNG format, with 3-channel RGB, 8-bit depth in each channel. There are 4 subtypes for benign cancer: Adenosis (A), Fibro Adenoma (FA), Tubular Adenoma (TA), and Phyllodes Tumor (PT). And there are 4 subtypes for malignant cancer: Ductal Carcinoma (DC), Lobular Carcinoma (LC), Mucinous Carcinoma (MC), and Papillary Carcinoma (PC). Please refer to Table 4 for the detailed information of this dataset BreaKHis is an imbalanced dataset, as almost 70% of the images representing malignant breast tumors. Some sample images of subtypes from the BreaKHis 40 × dataset is shown in Fig 5.

**Table 4:** Structure of the BreaKHis dataset.

Classes	Subtypes	Magnification Factors				Total
		40×	100×	200×	400×	
**Benign (B)**	Adenosis (A)	114	113	111	106	444
	Fibroadenoma (F)	253	260	264	237	1,014
	Tubular Adenoma (TA)	109	121	108	115	453
	Phyllodes Tumor (PT)	149	150	140	130	569
**Malignant (M)**	Ductal Carcinoma (DC)	864	903	896	788	3,451
	Lobular Carcinoma (LC)	156	170	163	137	626
	Mucinous Carcinoma (MC)	205	222	196	169	792
	Papillary Carcinoma (PC)	145	142	135	138	560
**Total**		1,995	2,081	2,013	1,820	7,909

### 4.2 Setups and evaluation metrics

Following the experimental protocal proposed in [4], we perform binary and multi-class classification experiments on each BreaKHis magnification factor. We repeat each experiment three times and report their average results. In each dataset, we applied the same data augmentation [32] techniques, including height and width shift with a factor of 0.125, a horizontal flip, and constant fill mode, as data augmentation is helpful for improving the accuracy of classification, preventing over-fitting and enhancing the robustness of the network. We used the down sampling method to convert the image size to 224 × 224 and normalized data with Zero-mean normalization [33]. The entire network is trained end-to-end by SGD [26] with back-propagation. We use SGD with a mini-batch size of 20, by using the momentum of 0.9 and a weight decay of 1e-4. All models are trained for 300 epochs from scratch. We initialize the weights according to the method in [34] and use the Softmax function for the final classification. The different settings for the binary and multi-class classification experiments are as followings:

(A) binary classification: we use the BHCNet-3 structure, and the BreaKHis dataset is randomly divided into 60% training set and 40% testing set for each magnification factor. We use four different learning rate scheduler methods: step scheduler, cosine scheduler, exponential scheduler, and Gauss error scheduler. For the step scheduler, it starts with a learning rate of 0.01, decrease it to 0.006, 0.001, 0.0001 after each of the 100th, 140th, 220th epoch. For the exponential scheduler initially we set the learning rate to 0.01, and *λ* = 0.98. For Gauss error scheduler, we set *lr*_*max*_ = 0.01, *lr*_*min*_ = 0.0001, we use *α* = −4, *β* = 4 to classify images with 40× magnification factor, and use *α* = −3, *β* = 3 to classify images with other magnification factors. For the cosine scheduler, we start with a learning rate of 0.01 and decrease it to 0.0001.
(B) multi-classification: we use the BHCNet-6 structure, and the BreaKHis dataset has been randomly dividing into 70% training set and 30% testing set for each magnification factor. we only use Gauss error scheduler to adjust the learning rate as follows: *lr*_*max*_ = 0.1, *lr*_*min*_ = 0.0001, *α* = −3, *β* = 3.

To evaluate the proposed models, we use the Scikit-learn [35] to obtain classificaiton performance, including AUC [36], Matthews Correlation Coefficient (MCC) [37], precision, recall, F-measure, and confuse matrix. Macro average is used for the final results of our experiment. The MCC index takes true and false positives and negatives into account,and it is generally regarded as a balanced measure which can be used even in the imbanlanced classification scenario. Since the number of images in each category of the BreakHis dataset is imbalanced, MCC index is used in our expeirment for measuring the performance of the models.

### 4.3 Binary classification results

**Fig 6:**
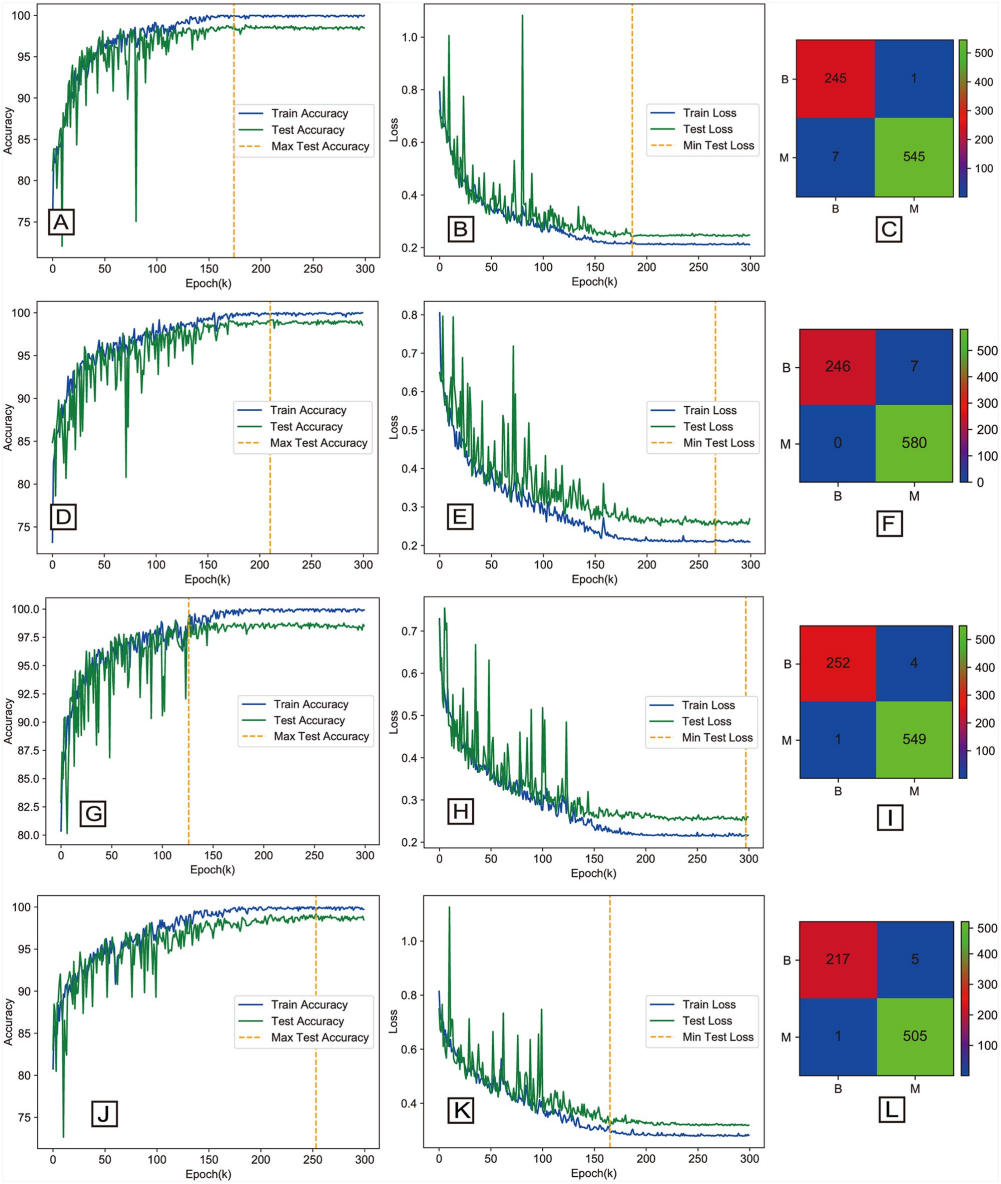
The accuracy curve and loss curve and confusion matrix of BHCNet-3 for the binary classification. The left column is the accuracy curve. The middle column is the loss curve, and the right column is the confusion matrix. From top to bottom are 40X, 100X, 200X and 400X magnification factors.

The experiment result measured in accuracy for binary classification is shown in Table 5. For images with magnification factors of 40×, 100×, 200×, and 400×, the classification accuracy is 98.87%, 99.04%, 99.34%, and 98.99%, respectively. The accuracy curve, loss curve and confusion matrix of the experiment result for each magnification factor is shown in Fig 6. We compared our method with some existing approaches that were reported for the BreaKHis dataset with the same dataset experiment setup (shown in Table 5). The evaluation metrics including AUC, MCC, recall, precision and F-measure values computed from the BHCNet-3 in each magnification factor is shown in Table 6. Now, we compare our methods and the results with the recent works.

**Table 5:** The accuracies performance of BHCNet-3 for the binary classification.

References	Methods	40×	100×	200×	400×
**Kahya et al. (2017)** [39]	ASSVM	94.97	93.62	94.54	94.42
**Wei et al. (2017)** [18]	BiCNN	97.89	97.64	97.56	97.97
**Pratiher et al. (2018)** [40]	L-Isomap and SSAE	96.8	98.1	98.2	97.5
**Bardou et al. (2018)** [4]	CNN	94.65	94.07	94.54	93.77
	CNN + Augmented	96.82	96.96	96.36	95.97
	SVM	92.71	93.75	92.72	92.12
	Ensemble CNN model	98.33	97.12	97.85	96.15
	BoW/DSIFT	66.72	69.06	62.42	52.75
	BoW/SURF	85.45	79.77	78.97	78.57
	LLC/DSIFT	72.74	78.04	78.97	75.00
	LLC/SURF	87.00	82.50	84.00	87.91
**Present Work**	BHCNet-3 + step	98.29±0.24	98.68±0.17	99.26±0.17	98.76±0.11
	BHCNet-3 + Cos	98.75±0.17	98.88±0.20	99.17±0.15	98.72±0.17
	BHCNet-3 + Exp	98.12±0.13	98.80±0.17	98.88±0.27	98.21±0.34
	BHCNet-3 + ERF	**98.87** ± **0.10**	**99.04** ± **0.10**	**99.34** ± **0.06**	**98.99** ± **0.17**

**Table 6:** The evaluation metrics computed from best result of the BHCNet-3 in each magnification factor and compare the result to the previous work.

References	magnification	AUC (%)	MCC (%)	Precision (%)	Recall (%)	F-Measure (%)
**Nahid et al.** [15]	40×	94.40	-	94.00	96.00	95.00
	100×	95.93	-	98.00	96.36	97.00
	200×	97.19	-	98.00	98.20	98.00
	400×	96.00	-	95.00	97.79	96.00
**Bardou et al.** [4]	40×	-	-	97.80	97.57	97.68
	100×	-	-	98.58	96.98	97.77
	200×	-	-	95.61	99.28	97.41
	400×	-	-	97.54	96.49	97.07
**present work**	40×	99.93	97.68	98.52	99.16	98.83
	100×	99.66	98.02	99.40	98.62	99.00
	200×	99.96	98.57	99.44	99.13	99.28
	400×	99.82	98.05	99.28	98.78	99.02

In [38], Chan et al. used the support vector machine to classify breast cancer tumors into benign and malignant and achieved the F-measure of 0.979 at 40 × magnification factor. In [14], Spanhol et al. used the pre-trained BVLC CaffeNet Model as a feature extractor, which is then used as input for logistic regression classifier trained to classify breast cancer tumors into benign and malignant. The authors reported an accuracy ranging from 86.7% to 88.8%. In [39], Kahya et al. proposed an efficient feature selection and classification of breast cancer histopathology images, which is based on the idea of sparse support vector machine combined with Wilcoxon rank sum test. Experimentally, the reported accuracy is ranging from 93.62% to 94.97%. In [18], Wei et al. proposed a BiCNN model and an advanced data augmented method. The BiCNN considered class and sub-class labels of breast cancer as prior knowledge, which can restrain the distance of features of different breast cancer pathological images. The BiCNN achieves accuracies about 97%. In [40], Pratiher et al. proposed a novel L-Isomap aided manifold learning & stacked sparse autoencoder framework for reliable and robust BC classification using HI’s. The obtained accuracies were 96.8%, 98.1%, 98.2%, and 97.5% for images with magnification factors 40 ×, 100 ×, 200 ×, and 400 ×, respectively. In [4], Bardou et al. compared two machine learning approaches for the automatic classification of breast cancer histology images. The first approach is based on the extraction of a set of handcrafted features encoded by two coding models (bag of words and locality constrained linear coding), and use these features for training support vector machines, which achieved classification accuracy between 72.74% and 87.91%. The second approach is based on the design of CNN, which achieved classification accuracy between 96.15% and 98.33%. Our proposed BHCNet-3 network outperforms the approaches in [4, 14, 18, 39, 40] in terms of accuracies by achieved the best accuracies between 98.87% and 99.34%.

### 4.4 Investigation of the parameters of the Gauss error scheduler

**Fig 7:**
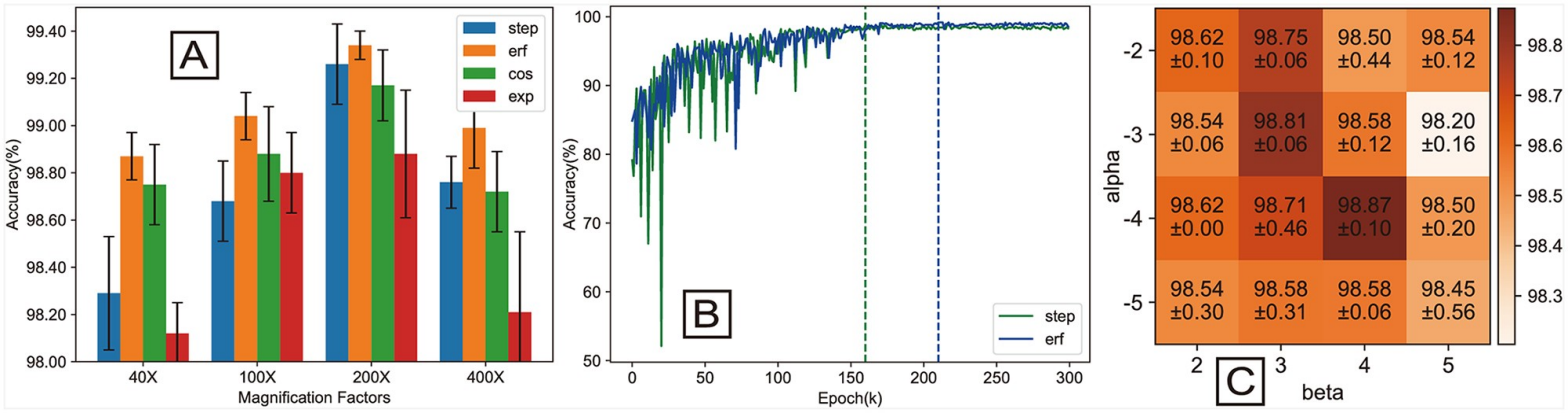
(A) Performance comparison of different learning rate schedulers. (B) Training curves of different learning rate scheduler in 100× magnification factor. (C) Confusion matrix for different *α* (y-axis) and *β* (x-axis) for the test accuracy.

The binary classification results show better accuracy when using Gaussian error scheduler compared with using step scheduler, cosine scheduler, and exponential scheduler. As it can be seen from Fig 7A, the accuracy performance of Gaussian error scheduler is better than the accuracy performance of others schedulers. We find that exponential scheduler is less accurate than other schedulers. In this condition, we think that the Gaussian error scheduler and step scheduler can make the model use the maximum learning rate and the minimum learning rate for stable training. This may be the key to get better performance. The training curves of step (green curve) and Gauss error (blue curve) scheduler for 100 × magnification factor are shown in Fig 7B. It can see from the training curve that Gauss error scheduler is more stable than step scheduler, and step scheduler is sharp shock before the 100th epoch. After 140th epoch, the step scheduler slowly and steadily converges, and the maximum accuracy is achieved in 160th epoch. After 180th epoch, the Gauss error scheduler steadily converges, and the maximum accuracy is achieved in 210th epoch. Although Gauss error scheduler takes a longer time than step scheduler to converge, Gauss error scheduler’s convergence is better than step scheduler. This has reached our goal which is described in Section 3.2.

We show a 4 × 4 confusion matrix for the *α* and *β* influence based on BHCNet’s performance on BreaKHis 40 × magnification dataset in Fig 7C. It can be seen from the confusion matrix that ERF(-3, 3) and ERF(-4, 4) achieve higher classification accuracy of 98.81% and 98.87%, respectively. According to the experimental results of Gauss error scheduler on Cifar and BreaKHis, we recommend that the parameters of Gauss error scheduler take *α* = −3, *β* = 3. In multi-classification experiments, we set the parameters of Gauss error scheduler as follows: *lr*_*max*_ = 0.1, *lr*_*min*_ = 0.0001, *α* = −3, *β* = 3.

### 4.5 Multi-classification results

**Fig 8:**
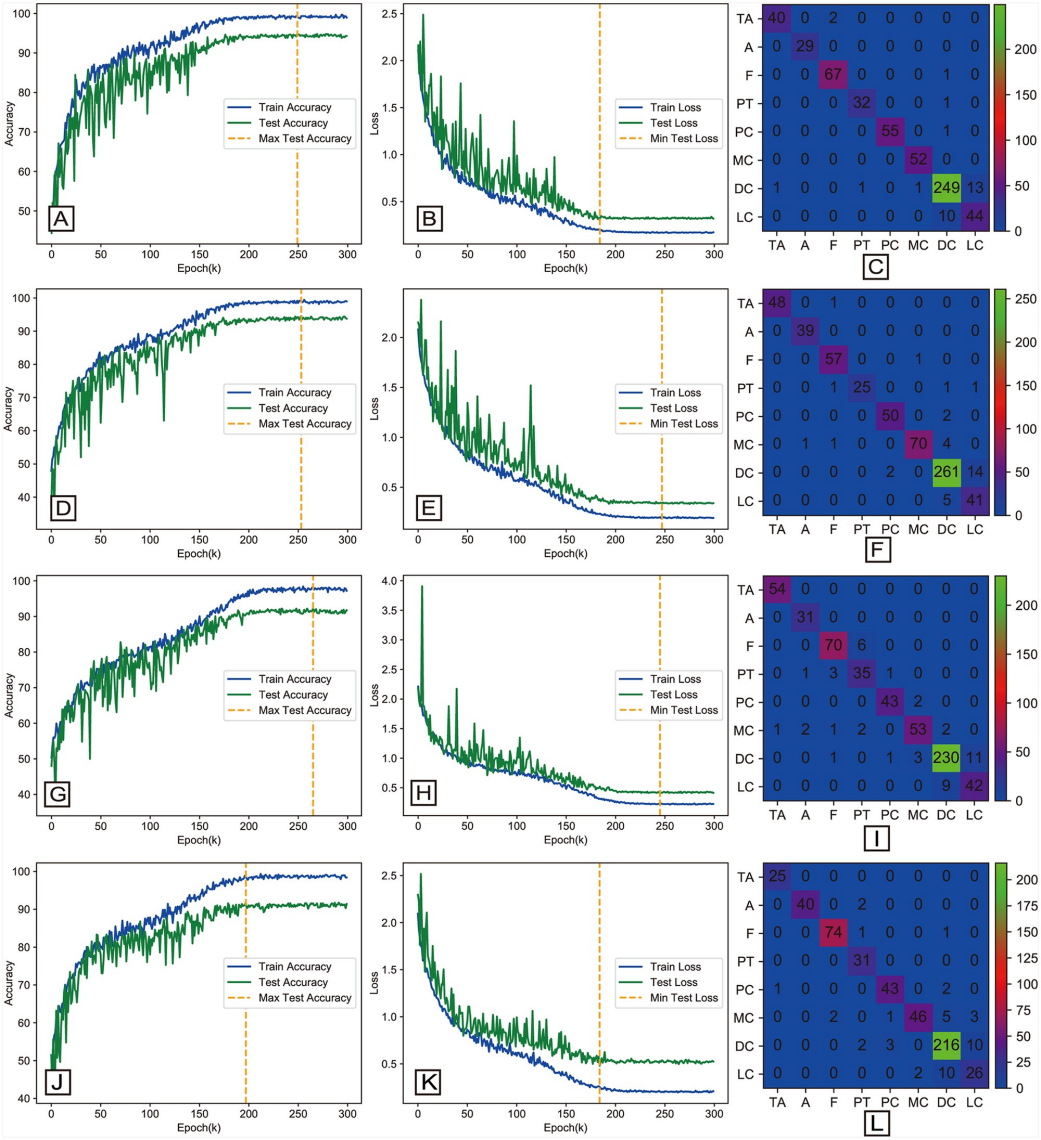
The accuracy curve and loss curve and confusion matrix of BHCNet-6 for the multi-classification. The left column is the accuracy curve. The middle column is the loss curve, and the right column is the confusion matrix. From top to bottom are 40X, 100X, 200X and 400X magnification factors.

All multi-classification accuracy results are given in Table 7. The obtained accuracies are 93.74%, 93.81%, 92.22%, and 90.66% for images with magnification factors 40×, 100×, 200×, and 400×, respectively. The accuracy curve, loss curve, and confusion matrix for multi-classification for each magnification factor are shown in Fig 8. The evaluation metrics including AUC, MCC, recall, precision and F-measure values computed from the best result of the BHCNet-6 for each magnification factor are shown in Table 8.

**Table 7:** The accuracies performance of BHCNet-6 for the multi-classification.

References	Methods	40×	100×	200×	400×
**Chan et al. (2016)** [38]	SVM	55.6	-	-	-
**Bardou et al. (2018)** [4]	CNN	86.34	84.00	79.93	79.74
	CNN + Augmented	83.79	84.48	80.83	81.03
	SVM	82.89	80.94	79.44	77.94
	Ensemble CNN model	88.23	84.64	83.31	83.98
	BoW/DSIFT	66.72	69.06	62.42	52.75
	BoW/SURF	41.80	38.56	49.75	38.67
	LLC/DSIFT	60.58	57.44	70.00	46.96
	LLC/SURF	80.37	63.84	74.54	54.70
**Present Work**	BHCNet-6 + ERF	**94.43** ± **0.28**	**94.45** ± **0.15**	**92.27** ± **0.08**	**91.15** ± **0.43**

**Table 8:** The evaluation metrics computed from best result of the BHCNet-6 in each magnification factor and compare the result to the previous work.

References	magnification	AUC (%)	MCC (%)	Precision (%)	Recall (%)	F-Measure (%)
**Han et al.** [41]	40×	92.8 ± 2.1	-	-	-	92.9
	100×	93.9 ± 1.9	-	-	-	88.9
	200×	93.7 ± 2.2	-	-	-	88.7
	400×	92.9.8 ± 1.8	-	-	-	85.9
**Bardou et al.** [4]	40×	-	-	84.27	83.79	83.74
	100×	-	-	84.29	84.48	84.31
	200×	-	-	81.85	80.83	80.48
	400×	-	-	80.84	81.03	80.63
**present work**	40×	99.76	93.18	95.25	95.55	95.39
	100×	99.78	92.85	94.51	94.64	94.42
	200×	99.51	90.29	90.71	92.24	91.42
	400×	99.30	89.27	90.74	91.09	90.75

We compare our results with some state-of-art algoritm for multi-classification on the BreakHis dataset (show in Table 7). In [38], Chan et al. used the support vector machine to classify breast cancer tumors into eight subtypes of benign and malignant and achieved an accuracy of 0.556 for 40 × magnification factor. In [4], Bardou et al. compared two machine learning approaches for the automatic classification of breast cancer histology images into benign and malignant cancer subtypes classification. The first method is based on a CNN topology and trained for 20,000 iterations with the classification accuracy being 86.34%, 84.00%, 79.83%, and 79.74% for images with magnification factors 40 ×, 100 ×, 200 ×, and 400 ×, respectively. After providing CNN with data augmentation, the algorihtm reaches accuracy of 83.79%, 84.48%, 80.83%, and 81.03% respectively. The CNN model ensemble method was applied for the multi-class classification and achieved an accuracy of 88.23%, 84.64%, 83.31%, and 83.98% respectively. The second method is based on the extraction of a set of handcrafted features encoded by two coding models (bag of words and locality constrained linear coding), and support vector machineswere traiend on these features. The algorithm achieved classification accuracy between 41.80% and 80.37%. Our proposed BHCNet-6 and Gauss error scheduler achieves the accuracy between 90.66% and 93.81%, which outperforms the approaches proposed by Bardou et al. [4] and Chan et al. [38] in terms of accuracy.

## 5 Conclusion

In this work, we design a new convolutional neural network, the Breast Cancer Histopathology Image Classification Network (BHCNet), for the classification of breast cancer histopathology images. We design a small SE-ResNet module with fewer parameters to reduce the training parameters of the model, and to reduce the risk of model over-fitting. Through experiments, we find that compared with the bottleneck SE-ResNet module and basic SE-ResNet module, the parameters of the small SE-ResNet module is reduced to 29.4% and 33.3%, respectively. Furthermore, we proposed Gauss error scheduler, a novel learning rate scheduler that free the user from fine-tuning the learning rate parameter for SGD algorithm. On Cifar and BreaKHis datasets, the performance of the Gauss error scheduler is better then the step scheduler, cosine scheduler and exponential scheduler. For the binary classification task, the BHCNet-3 outperform the approaches in [4, 14, 18, 38, 39], and achieved a performance between 98.87% and 99.34%. For the multi-classification task, the BHCNet-6 outperforms the approaches in [4, 38], and achieved a performance between 90.66% and 93.81%. In the future, we will study the problem such as cell overlap and uneven color distribution in the pathological images of breast cancer obtained from different staining methods.
